# Chemical crosslinkers enhance detection of receptor interactomes

**DOI:** 10.3389/fphar.2013.00171

**Published:** 2014-01-07

**Authors:** Brian A. Corgiat, Jacob C. Nordman, Nadine Kabbani

**Affiliations:** Department of Molecular Neuroscience, Krasnow Institute for Advanced Study, George Mason UniversityFairfax, VA, USA

**Keywords:** nicotinic acetylcholine receptor, chemical crosslinking, mass spectrometry, protein–protein interaction, signaling network, interactome

## Abstract

Receptor function is dependent on interaction with various intracellular proteins that ensure the localization and signaling of the receptor. While a number of approaches have been optimized for the isolation, purification, and proteomic characterization of receptor–protein interaction networks (interactomes) in cells, the capture of receptor interactomes and their dynamic properties remains a challenge. In particular, the study of interactome components that bind to the receptor with low affinity or can rapidly dissociate from the macromolecular complex is difficult. Here we describe how chemical crosslinking (CC) can aid in the isolation and proteomic analysis of receptor–protein interactions. The addition of CC to standard affinity purification and mass spectrometry protocols boosts the power of protein capture within the proteomic assay and enables the identification of specific binding partners under various cellular and receptor states. The utility of CC in receptor interactome studies is highlighted for the nicotinic acetylcholine receptor as well as several other receptor types. A better understanding of receptors and their interactions with proteins spearheads molecular biology, informs an integral part of bench medicine which helps in drug development, drug action, and understanding the pathophysiology of disease.

## CROSSLINKING IN THE ANALYSIS OF RECEPTOR INTERACTOMES: TO LINK OR NOT TO LINK?

To biochemically study receptor–protein interactions, one must be able to isolate receptors and their interactomes from the lipid plasma membrane. This process is challenging because of the need to keep diverse protein–protein interactions intact during the extraction and purification of the protein complex. Additionally, the structure and subcellular localization of the receptor in the membrane dictates the chemical conditions required for protein extraction (Thomas and McNamee, 1990). Large polypeptide membrane spanning receptors, such as ligand gated ion channels, demand strong detergent-based solubilization in order to ensure extraction of all receptor subunits. The stringency of these detergent conditions, however, can lead to a loss in numerous receptor–protein associations. Receptors that are embedded in lipid-rich and cholesterol heavy regions of the plasma membrane (such as rafts) require unconventional solubilization methods since these areas are resistant to detergents (Li et al., 2004; Sot et al., 2006). The isolation of membrane bound receptors and their interacting proteins is not trivial and requires extensive optimization on a receptor-by-receptor basis (Kalipatnapu and Chattopadhyay, 2005; Sarramegn et al., 2006).

Cell-based imaging strategies for the study of receptor–protein interactions, such as fluorescence resonance energy transfer (FRET), circumvent this problem by examining the interaction within an intact cell (Truong and Ikura, 2001). FRET analysis, however, only measures protein–protein interaction at relatively large distances [~100 Angstroms (Å)] and therefore may not be very informative about direct protein coupling (Pollok, 1999). Biochemical assessment of receptor–protein interaction using standard affinity purification methods alone also suffers from several drawbacks. First, the chemical stringency of the biochemical processing steps likely compromises and/or interferes with some receptor–protein interactions. Second, common affinity purification methods such as immunoprecipitation (IP) or pulldown assays heavily rely on the specificity of the antibody or capturing bait and may bias toward abundant proteins and stable protein–protein interactions. In the absence of stringent controls, standard IP experiments can produce substantial false positive results. Finally, current biochemical methods used to detect protein interactions lack cellular spatial specificity; consequently, when a true interaction is discovered the subcellular localization of the interaction is unknown.

**FIGURE 1:**
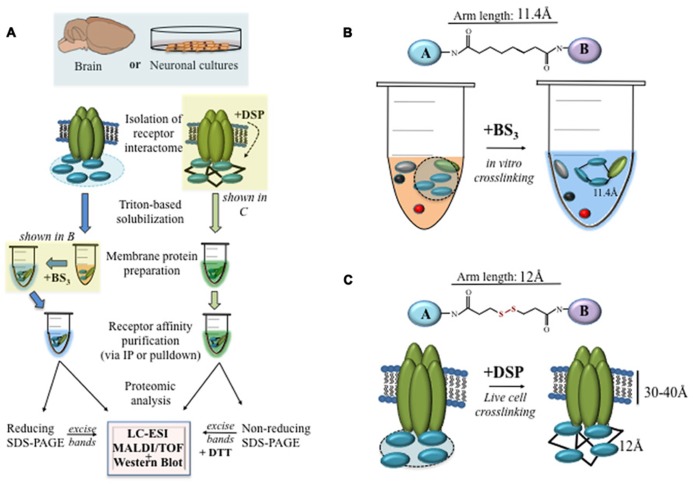
**Crosslinking the nAChR interactome. (A)** A flow chart showing the methods for isolation, crosslinking, and proteomic analysis of nAChR interactomes from brain tissue or neural cells. The experimental design should take into the consideration the choice of the crosslinker as well as the spacer arm. **(B)** The irreversible crosslinker BS_3_ is effective for crosslinking nAChR interactomes after membrane protein solubilization with Triton-X. **(C)** The membrane permeable crosslinker DSP, on the other hand, can be used to crosslink receptors and their interacting proteins in living cells.

New strategies have emerged for enhancing the detection of protein interactions. Methods such as protein fragment complementation and chemical crosslinking (CC) can stabilize transient or labile protein interactions *in vivo* and *in vitro* (**Box 1**), and therefore enable the identification of many proteins within the interactome (Kluger and Alagic, 2004; Morell et al., 2007). Conventionally CC has been used in the study of extracellular interactions of the receptor such as ligand binding (Gronemeyer and Govindan, 1986; Fanger et al., 1989; Boudreau et al., 2012; Kim et al., 2012), studies now reveal however a utility for cell permeable CC in the identification of the receptor interactome (**Figure 1A**; Guerrero et al., 2006; Nordman and Kabbani, 2012). In particular, dynamic changes in protein–protein associations within receptor interactomes appear better detected by CC at various stages of the receptor preparation and purification method (Vasilescu et al., 2004). Interactions that are generally too weak or too transient to be discovered in standard pulldown or IP assays alone, can be stabilized by covalent crosslinkers during the membrane solubilization process (Bond et al., 2009; Nordman and Kabbani, 2012). The common use of stringent chemical detergents such as radio-immunoprecipitation assay (RIPA) buffers, which interfere with many types of protein–protein interactions, can also benefit from the addition of covalent crosslinkers which are generally unperturbed by the RIPA reagent. Moreover, CC can be effectively combined with affinity purification protocols such as the IP prior to the mass spectrometry analysis (Vasilescu et al., 2004). To eliminate non-specific interactions of proteins during CC, the assay requires optimization before the start of the study. It is also not uncommon to run non-crosslinked samples in parallel during the course of a study (Kim et al., 2012).

**Box 1. Technical Toolbox.**

**FIGURE 2:**
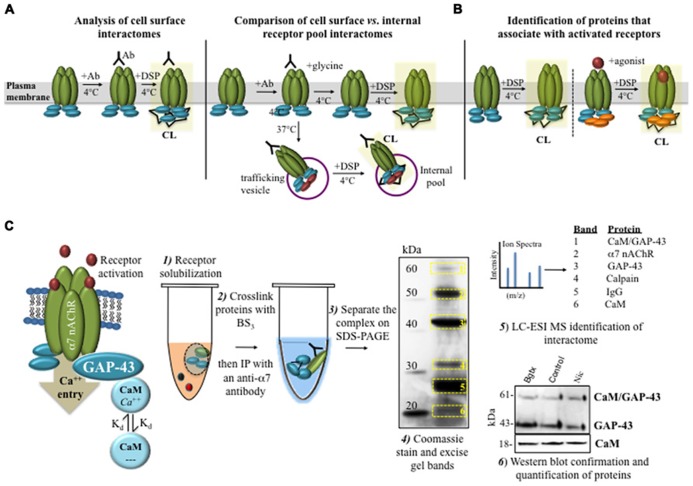
**Identifying protein interactions that mediate nAChR trafficking, localization, and signaling. (A)** Cell surface receptors can be selectively labeled with an anti-α7 nAChR monoclonal Ab. Cell surface labeling (at 4°C) can be combined with DSP in order to crosslink the receptor interactome. Alternatively, antibody labeling and crosslinking can be used to examine changes in the receptor interactome between internalized and cell surface nAChRs. **(B)** Chemical crosslinking can be used to study the dynamics of nAChR–protein interactions under various ligand treatment conditions. **(C)** Experimental evidence on α7 nAChR interactions with GAP-43 and CaM in developing neural cells. BS_3_ was used to crosslink the α7 nAChR interactome from differentiating cells. An IP was utilized to purify the receptor, which was visualized by SDS-PAGE. Protein identity was confirmed using LC-ESI MS and Western blot. These experiments demonstrate dynamic changes in CaM/GAP-43 association with α7 nAChR in response to nicotine activation (Nordman and Kabbani, 2012).

• To crosslink solubilized membrane proteins *in vitro* with BS_3_, add 2 mM BS_3_ to the enriched receptor fraction for 2 h at 4°C and mix (**Figure 1B**).

• To crosslink proteins *in vivo*, add 2.5 mM DSP to cultured cells for 2 h at 4°C (**Figure 1C**). The chemical reaction with DSP can be terminated by the addition of 50 mM Tris-HCI (pH 7.5) at 4°C.

• Clever use of DSP crosslinking enables a range of experiments on receptor–protein interactions including an analysis of receptor interactomes at the cell surface, inside recycled vesicles, or in response to ligand stimulation (**Figures 2A,B**).

• A crosslinked receptor interactome can be purified using standard methods for immunoprecipitation and mass spectrometry (**Figure 2C**).

A number of crosslinkers have been used to study receptor–protein interactions in cells (Brenner et al., 1985; Shinya et al., 2010; Miteva et al., 2013). These compounds are characterized by differences in their spacer arm as well as the composition of the two amine binding groups that recognize and covalently bind specific functional groups on target proteins (**Figures 1B,C**; Sinz, 2003; Trakselis et al., 2005). **Table 1** lists crosslinkers that have been used to study receptor binding to intracellular proteins. The choice of a spacer arm length, between 5 and 25 Å, is experimentally important because it enables the identification of receptor–protein interactions at specific distances. A recent study utilized agarose beads whose surface was covalently linked with a cleavable chemical crosslinker by spacers of varied lengths to study the interactome of the post-synaptic density isolated from the rodent cortex (Yun-Hong et al., 2011). Experiments successfully demonstrate assemblages of proteins at various subcellular distances and compartments, including the post-synaptic density, thus underscoring the utility of the approach in the characterization of interactomes based on spacer arm properties. However, a possible disadvantage of CC is that some antibodies are no longer able to recognize their target protein after crosslinking (Boudreau et al., 2012).

**Table 1:** A summary of crosslinkers used in the analysis of receptor protein interactions.

Chemical name^1^	Membrane permeable	Reversible	Reactive toward	Receptor interactome applications	Spacer arm
DSS	Y	N	Amines	Gardoni etal. (2002), Wang etal. (2002),Chu etal. (2004)	11.4
DSP	Y	Y; by DTT	Amines	Luttrell etal. (1999), Lau and Hall (2001), Quian etal. (2001), Shenoy etal. (2006)	12.0
BS_3_	N	N	Amines	Aldecoa etal. (2000), Nordman and Kabbani (2012)	11.4
*MBP	Y	N	Sulfydryls	–	<5
*ANB-NOS	Y	N	Amines	–	7.7
*Sulfo-SAED	N	Y; by DTT	Amines	Yun-Hong etal. (2011)	23.6

*Denotes photo-reactive linkers.

1 IUPAC Names: DSS, disccinimidyl suberate; DSP, dithiobis(succinimidyl propionate; BS_3_, bis(sulfosuccinimidyl) suberate; MBP, 4-maleimidobenzophenone; ANB-NOS, N-5-azido-2-nitrobenzoyloxysuccinimide; SAED, sulfosuccinididyl 2-(7-azido-4-methylcoumarin-3 -acetoadmio)-ethyl-1,3 ′-dithiopropionate.

## CROSSLINKING ENABLES DETECTION OF PROTEINS THAT BIND THE RECEPTOR IN VARIOUS STATES

Interaction with trafficking and chaperone proteins is important for directing the localization and function of the receptor (Jeanclos et al., 2001; Lin et al., 2002; Xu et al., 2006; Kabbani et al., 2007; Nordman and Kabbani, 2012; Colombo et al., 2013). Protein associations may also contribute to receptor conformation at the cell surface (Giniatullin et al., 2005). While the ability to detect receptors at the plasma membrane and in the cytosol has been traditionally reliant on epitope tagging, live cell stain, and cell surface labeling methods such as biotinylation, CC has emerged as a complementary tool in the study of interacting proteins responsible for receptor cellular trafficking and localization. Numerous examples exist in the literature, however, work on β2 adrenergic receptor internalization has been central to understanding mechanisms in GPCR internalization (Lefkowitz and Shenoy, 2005). Key findings on β2 adrenergic receptor internalization have come from experiments using various crosslinkers to detect the association of the receptor with the endocytosis machinery of the cells. First, IP was used in conjunction with DSP [dithiobis(succinimidyl propionate)] crosslinking in human embryonic kidney (HEK) 293 cells to quantify changes in β2 adrenergic receptor internalization and demonstrate that the internalized receptor was bound to β-arrestin, which functions as an endocytic adaptor of the receptor complex (Shenoy et al., 2006). In a second similar study, also performed in HEK 293 cells that stably express the β2 adrenergic receptor, DSP was used to show that the β2 adrenergic receptor binds the trafficking and regulatory proteins β-arrestin and c-Src in its “desensitized” state (Luttrell et al., 1999). The study interestingly demonstrates that receptor signaling is sustained even in the absence of ligand binding. A third study on β2 adrenergic receptor internalization employed covalent protein crosslinking with DSP for the detection of transient, agonist-promoted association of dynamin and c-Src, showing how these protein interactions can alter the rate of receptor internalization in the cell (Ahn, 1999).

Crosslinking has also enabled detection of changes in receptor function for the angiotensin receptor (Quian et al., 2001) and has been useful in determining interactions impacted by post-translational modification (Cao et al., 1999; Connolly, 1999; Ehlers, 2000). When combined with cell surface labeling, CC has been effective in determining changes in receptor glycosylation. Differential glycosylation of cell surface human and rat (r) calcitonin (CT) receptor-like receptors (CRLR) as a result of interactions with accessory receptor activity-modifying proteins (RAMPs)-1 or -2 was confirmed by CC using BS_3_ [bis(sulfosuccinimidyl) suberate] in *Drosophila* S2 cells (Aldecoa et al., 2000). In this study CC revealed receptor components with the size of rCRLR, increased by the molecular weights of the corresponding RAMP – suggestive of a direct association between the receptor and the accessory protein during ligand activation.

## NICOTINIC RECEPTOR INTERACTOMES DEFINED BY CROSSLINKING

Nicotinic acetylcholine receptors (nAChRs) are a family of ligand gated ion channels expressed throughout the nervous system contributing to learning, memory, and goal driven behavior (Changeux, 2012). Recent evidence also reveals that nAChRs operate by coupling to intracellular proteins such as heterotrimeric G proteins (Kabbani et al., 2013). Chronic nicotine exposure gives rise to neural adaptations such as an up-regulation of specific nAChRs through cell-delimited post-translational mechanisms (Sallette et al., 2005; Colombo et al., 2013). These receptor mechanisms are a hallmark of nicotine addiction yet it is still unclear which signaling pathways and mechanism regulate nAChR assembly and trafficking inside the cell. Proteomic studies, based on yeast-two-hybrid as well as conventional IP experiments have led to the identification of several intracellular proteins that bind nAChR subunits in the brain (Kabbani et al., 2007; Paulo et al., 2009; Nordman and Kabbani, 2012; McClure-Begley et al., 2013). Directed protein interaction screens have also enabled discovery of proteins responsible for nAChR trafficking and assembly (Lin et al., 2002; Lansdell et al., 2005; Kabbani, 2008; Rezvani et al., 2009).

In the hippocampus, α7 nAChRs are expressed pre- and post-synaptically, contributing to GABA and glutamate neurotransmission (Liu et al., 2006; Lozada et al., 2012). α7 receptors are also found to mediate the growth of axons (Hancock et al., 2008; Nordman and Kabbani, 2012) and dendrites (Campbell et al., 2011) in the developing hippocampus. Using the membrane impermeable and irreversible crosslinker BS_3_, we have defined dynamic changes in α7 interaction within solubilized membrane fractions from differentiated PC12 cells and hippocampal neurons (**Figure 1B**; Nordman and Kabbani, 2012). We show that α7 receptors are directly coupled to a G protein pathway consisting of Gαo, Gprin1, and GAP-43 in growing cells (Nordman and Kabbani, 2012; **Figure 1C**). In these studies CC was vital to the detection of changes in receptor interaction with signaling molecules and heterotrimeric G proteins. The CC method was also able to enhance the detection of small signaling molecules such as receptor kinases in both Western blots and mass spectrometry experiments (Hu et al., 2010; Nordman and Kabbani, 2012). For example, using BS_3_ to crosslink the α7 nAChR network after nicotine activation, we identified rapid changes to the calcium-mediated signaling pathway of the receptor, which consisted of a dynamic association between GAP-43 and calmodulin (CaM) in the growing neurite (**Figure 2C**; Nordman and Kabbani, 2012). In particular, activation of the α7 nAChR was found to promote a rapid association between the receptor and CaM bound GAP-43. This interaction was rapidly reversed by ligand inactivation of the α7 nAChR, showing that receptor association with CaM bound GAP-43 was driven by nAChR channel function and calcium elevation in the cell (Nordman and Kabbani, 2012). These findings on dynamic associations of CaM and GAP-43 within the α7 nAChR interactome could not have been detected using standard IP assays alone underscoring the utility of the crosslinker in the study of protein interactions under physiological conditions. In a similar study, CC with disuccinimidyl suberate (DSS) was used in identifying dynamic changes in calcium bound CaM kinase II and subunits of the NMDA glutamate receptor within the post-synaptic density of hippocampal neurons (Gardoni et al., 2002), thus underscoring the utility of the method in the study of rapid calcium driven changes in protein coupling in cells.

## LOOKING AHEAD

Proteomic and yeast-two-hybrid studies on receptor interactions have enabled a broad understanding on the diversity and function of receptor–protein interactions in cells. These studies have enabled an interaction-based framework for defining the mechanisms of receptor signaling. Receptor–protein interaction identification however is not sufficient for understanding how receptors operate in cells. In particular, important questions remain on the spatial specificity and temporal aspects of receptor expression and signaling in cells. For multi-subunit channel receptors such as the glutamate AMPA receptor, the addition of the membrane impermeable linker BS_3_ has proven effective in the analysis of receptor subunit composition at the cell surface (Boudreau et al., 2012). Similar approaches with the aim of detecting protein–protein interaction in living cells are now necessary. Advancement in the design and experimental utility of CC such as photo-reactive amino acid analogs (Suchanek et al., 2005) promises to enhance the study of receptor–protein interactions *in vivo*.

## Conflict of Interest Statement

The authors declare that the research was conducted in the absence of any commercial or financial relationships that could be construed as a potential conflict of interest.
